# Dyslipidemic Diet Induces Mobilization of Peripheral Neutrophils and Monocytes That Exacerbate Hemorrhagic Brain Injury and Neuroinflammation

**DOI:** 10.3389/fncel.2020.00154

**Published:** 2020-06-08

**Authors:** Xiuping Li, Xiaojing Cheng, Xuejiao Wang, Qiang Liu, Hongshan Ma, Minshu Li

**Affiliations:** ^1^Department of Neurology, Tianjin Neurological Institute, Tianjin Medical University General Hospital, Tianjin, China; ^2^Key Laboratory of Post-neurotrauma Neuro-repair and Regeneration in Central Nervous System, Tianjin Neurological Institute, Ministry of Education, Tianjin, China; ^3^Center for Neurological Diseases, The Third People’s Hospital of Datong, Shanxi, China; ^4^China National Clinical Research Center for Neurological Diseases, Beijing Tiantan Hospital, Capital Medical University, Beijing, China; ^5^Advanced Innovation Center for Human Brain Protection, Capital Medical University, Beijing, China; ^6^Beijing Key Laboratory of Translational Medicine for Cerebrovascular Disease, Beijing, China

**Keywords:** intracerebral hemorrhage, NAFLD, neuroinflammation, myeloid cells, immune response

## Abstract

**Background**: Non-alcoholic fatty liver disease (NAFLD) is a common liver condition characterized by a significant accumulation of lipids in the liver without excessive alcohol consumption. Accumulating evidence suggests a significantly increased risk of intracerebral hemorrhage (ICH) in NAFLD patients. However, it remains poorly understood whether and how NAFLD affects the outcome of hemorrhagic brain injury. Here, we examined the effects of diet-induce NAFLD on ICH injury and neuroinflammation in mice.

**Methods**: NAFLD was induced in C57BL/6 mice by feeding with a methionine-choline deficient (MCD) diet for 4 weeks. Collagenase and autologous blood models were used to evaluate the effects of NAFLD on ICH injury and neuroinflammation.

**Results**: MCD diet for 4 weeks induces NAFLD and hyperlipidemia in mice. Mice receiving the MCD diet have aggravated neurological deficits and brain edema after ICH. The augmentation of ICH injury was accompanied by brain infiltration of neutrophils and monocytes and increased production of pro-inflammatory factors. Before ICH, MCD diet-induced mobilization of neutrophils and monocytes in the periphery. Notably, the detrimental effects of NAFLD on ICH injury was ablated in mice receiving antibody depletion of neutrophils and monocytes.

**Conclusions**: These results suggest that NAFLD exacerbates neuroinflammation and ICH injury.

## Introduction

Intracerebral hemorrhage (ICH) is a severe type of stroke without effective treatment (Aronowski and Zhao, 2011). Epidemiological studies have identified that environmental and dietary factors are related to the incidence and outcome after ICH. Among factors that increase the risk of ICH, non-alcoholic fatty liver disease (NAFLD) is a common liver disease that affects up to 30% of the general population (Tsochatzis and Newsome, 2018). NAFLD is characterized by excessive deposition of lipids in liver parenchyma independent of heavy alcohol consumption (Kim et al., 2011; Younossi et al., 2018; Khoury et al., 2019). Accompanying the accumulation of hepatic fat, patients usually display insulin resistance, dyslipidemia, and escalated systemic inflammation (Gluchowski et al., 2017; Fiorucci et al., 2018). However, it remains elusive whether and how NAFLD affects ICH outcome.

Emerging evidence has demonstrated inflammation as a key element that contributes to secondary brain injury after ICH (Zhou et al., 2014). Following the ictus of ICH, blood components including leukocytes surge into the brain and activate resident glia and recruit immune cells such as neutrophils and monocytes (Mracsko et al., 2014; Sheth and Rosand, 2014; Zhang Z. et al., 2017). These inflammatory events accelerate the development of perihematomal edema and neurological deterioration (Haukeland et al., 2006; Farrell et al., 2012; Gao and Tsukamoto, 2016). Clinical studies have indicated that patients with NAFLD are characterized by an augmentation of systemic inflammation characterized by increased mobilization of circulating inflammatory cells, suggesting that NAFLD possibly induces mobilization of peripheral immune cells. Yet the influences of such mobilization on the pathological responses to ICH remain unknown. To address this question, we induced NAFLD by feeding mice with a methionine-choline deficient (MCD) diet and investigated the effects of NAFLD on hemorrhagic injury and neuroinflammation using two ICH models.

## Materials and Methods

### Animals

Experimental protocols were approved by the Institutional Animal Care and Use Committees of Tianjin Medical University General Hospital. All experiments were designed, conducted, and reported according to the ARRIVE (Animal Research: reporting of *in vivo* Experiments) guidelines. Male C57BL/6 mice (12 weeks old) were purchased from the Vital River Corporation (Beijing, China). Mice were housed in pathogen-free conditions with free access to food and water. All Surgeries on mice were performed under anesthesia. All mice were randomly assigned to each experiment.

### Induction of ICH in Mice

As we previously reported, ICH was induced in mice by injection of autologous blood or bacterial collagenase (Li et al., 2017a,b; Ren et al., 2018). First, mice were anesthetized with an intraperitoneal injection of ketamine (100 mg/kg) and xylazine (10 mg/kg). After placing the mice on a stereotactic frame, a 1-mm burr hole was drilled on the right side of the skull (2.3 mm lateral to the midline, 0.5 mm anterior to the bregma). For the collagenase ICH model, 0.0375U bacterial collagenase (Type IV-S, Sigma, St. Louis, MO, USA) dissolved in 0.5 μl saline was infused at the caudate nucleus (3.7 mm depth beneath the skull) through an infusion pump (Kd Scientific Inc., Holliston, MA, USA) at a rate of 0.5 μl/min. In some experiments, we induced the mouse ICH model by infusion of autologous blood using a double-injection method. Whole blood (30 μl) was withdrawn from the angular vein and then infused into the brain as previously described (Sun et al., 2016). 5 μl of blood was first injected to generate a clot at a depth of 3 mm beneath the hole. Then the needle was moved to a depth of 3.5 mm to inject the remaining 25 μl of blood at the rate of 1 μl/min. After surgery, animals were placed in cages and provided with free access to food and water.

### Study Design and Drug Administration

A total of 207 male C57BL/6 mice were used in this study. Mice were randomly assigned to each group according to the type of chow they were given. A methionine-choline deficient (MCD) diet (H10401, Beijing HFK Bioscience Company Limited) was fed for 4 weeks to produce the animal model of NAFLD before ICH induction. Mice fed the normal diet were used as controls. This administration persisted at the end of the experiment (Larter and Yeh, 2008; Zhang et al., 2014). The mouse ICH model was induced by infusion of collagenase or autologous blood at day 0. A series of assessments were performed at indicated time points after ICH. For the Gr-1^+^ cell depletion experiment, an anti-mouse Gr-1 monoclonal antibody (MAb-RB6-8C5; BioXcell, West Lebanon, NH, USA) was delivered by intraperitoneal injection 1 day before and 1 day after ICH induction at a dose of 250 μg per mouse (Condamine et al., 2014; Wang et al., 2015). More than 90% of Gr-1^+^ cells were depleted in mice receiving an anti-mouse Gr-1 monoclonal antibody. All data were analyzed by independent investigators blinded to the group assignment.

### Behavioral Assessment

Behavioral assessment was performed at indicated time points after ICH surgery to assess the motor, sensory, reflex, and balance functions. The modified Neurological Severity Score (mNSS), corner turning test, together with the foot-fault test were conducted as previously described (Clarkson et al., 2010; Klebe et al., 2017; Ren et al., 2018). The range of scores for mNSS is from 0 to 18 and mice were given 1 point for the inability to fulfill a task. The corner turning test was used to assess sensorimotor and postural asymmetries. Briefly, each mouse freely proceeds into a corner with an angle of 30 degrees and then turns right or left to exit. This task was repeated 10 times with at least 30 s intervals between trials. The percentage of right turns was calculated and recorded. For the foot-fault test, mice were placed on a grid device measuring 32 cm × 20 cm × 50 cm (length × width × height) with 12-mm mesh and allowed to roam freely for 5 min. A foot fault was defined as the mouse dropping its limb into the grid hole or resting with the grid at the wrist level. The percentage of foot faults was calculated according to the formula: foot faults/(foot fault + nonfoot fault steps) × 100.

### Brain Water Content Measurement

Brain water content was assessed on day 3 after ICH induction, as previously described (Han et al., 2017). Briefly, mice brains were removed without perfusion and dissected into three parts: the ipsilateral, the contralateral, and cerebellum. The collected tissues were weighed immediately to obtain wet weights and then dried for 24 h at 100°C to get dry weights. The percentage of water content was calculated using the formula: (wet weight-dry weight)/wet weight × 100%.

### Flow Cytometry

At days 3 after ICH, the brain, spleen, and peripheral blood of mice were obtained for flow cytometry analysis to detect the counts of immune cells and cytokine production. Single-cell suspensions were prepared as previously described (Jin et al., 2016; Liu et al., 2016; Yang et al., 2019). Briefly, mice were perfused with cold PBS, thereafter mouse brain tissues were harvested and digested with collagenase IV to form a single cell suspension. After the myelin was removed using a 30% Percoll solution (GE Healthcare Bio-Science AB, Uppsala, Sweden), cell pellets were harvested on the bottom of the tube and suspended in 1% BSA solution for staining. To harvest splenic cells, the spleen tissues were removed and pressed through a 70 μm nylon cell strainer in PBS. After lysis of red blood cells, cell pellets were prepared for staining. Blood samples were collected from mice by eyeball extirpating and were subjected to red blood cell lysis. Cell pellets were harvested and collected for staining. All antibodies used were purchased from Biolegend (San Diego, CA, USA) unless otherwise indicated. The following antibodies were used in this study: CD45 (30-F11), CD11b (M1/70), CD3 (145-2C11), CD4 (GK1.4), CD8 (53-6.72), CD19 (1D3), Ly6C (HK1.4), Ly6G (1A8). Rabbit anti-MMP-9 (ab38898) antibody or Rabbit IgG, polyclonal - Isotype Control (ab37415) was purchased from Abcam (Cambridge, MA, USA), and Alexa Fluor^®^488-conjugated goat anti-rabbit IgG (H+L) was used as the secondary antibody (ab150077, Abcam). Meanwhile, the isotype controls were stained, respectively. The staining process followed the protocol in the manual and flow cytometry data were obtained using a FACS Aria III (BD Bioscience, San Jose, CA, USA). The data were analyzed by Flow Jo version 7.6.1 (flowjo.com). For the procedure for absolute counting of cells using flow cytometry, we adopted two methods to count interested cell populations in flow cytometry. One is that we recorded the volume of cells that have passed through the flow. The gate on the interested population and work out the concentration of cells (events recorded/volume recorded). With that concentration and a defined volume of cells to begin with we can work out the absolute cell number from the original volume (= concentration × volume). The other one is that we counted the total number of single cells harvested from the brain or spleen by trypan blue method. After staining, then we run and gate our interested cell population using flow cytometry. The formula we calculated cell number is the total number multiplied by our interested cell population step by step.

### Real-Time Polymerase Chain Reaction

Three days after ICH induction, total RNA was extracted from the ipsilateral hemisphere of brain tissue using Trizol reagent (Invitrogen, Carlsbad, CA, USA) according to the manufacturer’s instructions. The concentration of RNA was measured by ultraviolet spectrophotometry at 260/280 nm. Then total RNA was reverse transcribed into cDNA utilizing the PrimeScript™ RT reagent Kit (TaKaRa, Shiga, Japan). All procedures were conducted as instructed. SYBR Green PCR Master Mix (Roche, Indianapolis, IN, USA) was used to amplify the gene sequences on the Opticon 2 Real-Time PCR Detection System (BioRad, Hercules, CA, USA). The primers used in this experiment are listed in Supplementary Table S1. GAPDH served as a reference gene and mRNA expression was calculated as fold changes using the 2^−ΔΔCt^ method. The primer sequences used for RT-PCR analysis in this study were listed in Supplementary Table S1.

### Assessment of BBB Permeability

On day 3 after ICH, BBB permeability was measured by quantification of Evans Blue (Sigma, St. Louis, MO, USA) extravasation, as previously described (Zhang Y. et al., 2017; Ren et al., 2018). Evans Blue (2% in saline, 4 ml/kg) was injected through the caudal vein 2 h before the brain was collected. the mice were anesthetized with 10% chloral hydrate and perfused with PBS. After decapitation, the brains were removed and slabbed. Tissues were then photographed for qualitative analysis. For quantitative analysis, the brain tissues were removed, weighed, and placed in dimethylformamide. After homogenization, the mixture was incubated for 72 h in a 60°C water bath and centrifuged at 1,500 *g* for 10 min. The absorbance value of supernatant was measured by the fluorescence spectrophotometer. The tissue content of Evans Blue was quantified from a linear standard curve.

### Hematoxylin & Eosin (H&E) Stain, Oil Red O Stain

After mice received an MCD diet or control diet for 4 weeks, liver tissues were harvested and fixed using 4% paraformaldehyde. Then, 8 μm-thick sections were prepared with a microtome (Leica Biosystems, Nussloch, Germany). After dewaxing and rehydrating, the tissue sections were stained with H&E using a commercial kit (Solarbio, Beijing, China). Microscopic images were obtained using a microscope (Olympus, Tokyo, Japan). For Oil Red O stain, the fixed liver tissues were embedded with OCT and sliced into 8 μm-thick sections. After washing in distilled water, slices were soaked in 60% isopropyl alcohol for 20-30 s. Then, the slices were placed in a dyeing tank filled with a modified oil red O staining solution (G1261, Solarbio Life Sciences) and stained for 10–15 min in the dark. After separating and washing, the slices were counterstained with hematoxylin solution for 1–2 min to reveal the nuclei. Finally, slices were mounted with the glycerin gelatin for observation.

### Assessment of Triglyceride (TG)

Lipids in the serum and liver were measured according to the manual of the triglyceride assay kit (A110-2-1, Nanjing Jiancheng Bioengineering Institute, Nanjing, China). Triglyceride (TG) from serum and liver samples was estimated by enzymatic colorimetric GPO-PAP method. The whole blood was centrifuged to separate serum. For the liver tissue, the supernatant was centrifuged and collected after homogenization. The sample was mixed with the working solution and incubated for 10 min at 37°C. The absorbance was measured at 510 nm. The contents of triglyceride were measured by calculating the absorbance value of the standard and the sample.

### Coagulation Time and Bleeding Time Measurement

To assess the coagulation time of mice, blood was acquired from the angular vein using glass capillary after anesthesia. Then the tube filled with blood was put on a flat top. Equal length of the capillary tube was snapped every 30 s until a fibrin thread formed. As for the bleeding time of mice, we used a scalpel blade to transect the tail starting from 2 mm off the tip. The tail was immersed in a clear tube filled with saline at 37°C. The bleeding time was defined as time to cessation of bleeding. The maximum observing time was 20 min. If there was no bleeding within 30 s, it was considered as stopped.

### Statistical Analysis

The sample size was determined by power analysis adopting a significance level of α = 0.05 with 80% power to assess significant differences. We performed the power analysis and sample size calculations using SAS 9.1 software (SAS Institute Inc., Cary, NC, USA). Data were analyzed by at least two investigators blinded to group assignment. All values are shown as mean ± SD. Statistical analyses were conducted using Graphpad 6.0 software. The unpaired 2-tailed Student’s *t-*test was used for determining the significance of differences between the two groups and the survival index was analyzed by Log-rank (Mantel-Cox) test. *P* < 0.05 is considered significant.

## Results

### NAFLD Exacerbates Brain Injury and Neurological Outcome After ICH

**Figure 1:**
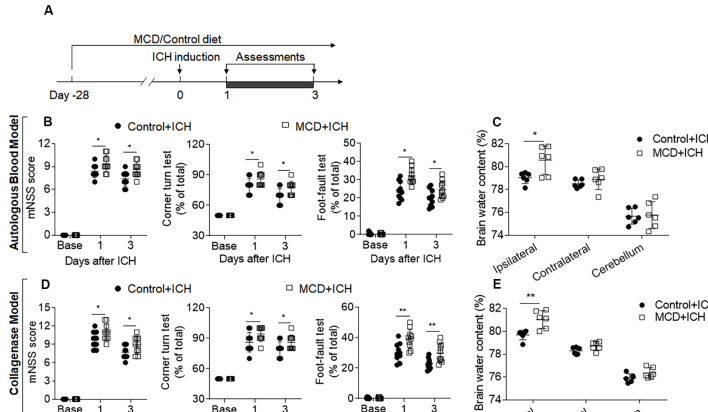
Non-alcoholic fatty liver disease (NAFLD) aggravated neurological deficits and brain edema in intracerebral hemorrhage (ICH) mice. **(A)** Flow chart illustrates the individual diet administration and experimental design. Mice were fed the methionine-choline deficient (MCD) diet for 4 weeks to develop the NAFLD model, and this administration persisted to the end of the experiment. ICH was induced in C57BL/6 mice by injection of autologous blood or collagenase at day 0. Neurological deficits and brain water content were measured at days 1 and 3. **(B,D)** The statistical data of mNSS, Corner turning test, and foot-fault test in ICH mice with control diet or MCD diet at baseline, days 1 and 3 after injection of autologous blood **(B)** or collagenase **(D)**. *n* = 10 mice per group. **(C,E)** The Brain water content in mice with the control or MCD diet in autologous blood model **(C)** or collagenase model **(E)** on day 3 after ICH. *n* = 6 mice per group. Data are presented as mean ± SD. *P < 0.05, **P < 0.01.

To determine the impact of NAFLD on brain injury after ICH, we examined neurological deficits and brain water content in ICH mice receiving an MCD diet or normal control diet. Before ICH induction, mice were fed with an MCD diet or control diet for four consecutive weeks (Figure 1A). After 4 weeks of MCD diet, dramatic lipid accumulation in the liver and hyperlipidemia were observed while coagulation time remained unaltered (Supplementary Figure S1). After ICH surgery, these mice continued to receive an MCD diet or control diet until the end of experiments (Figure 1A). We found that NAFLD mice receiving MCD diet had significantly increased neurological deficits and brain water content at days 1 and 3 after ICH induction, as compared to control mice (Figures 1B–E), suggesting that NAFLD aggravates neurological dysfunction and brain injury in the acute stage of ICH.

**Figure 2:**
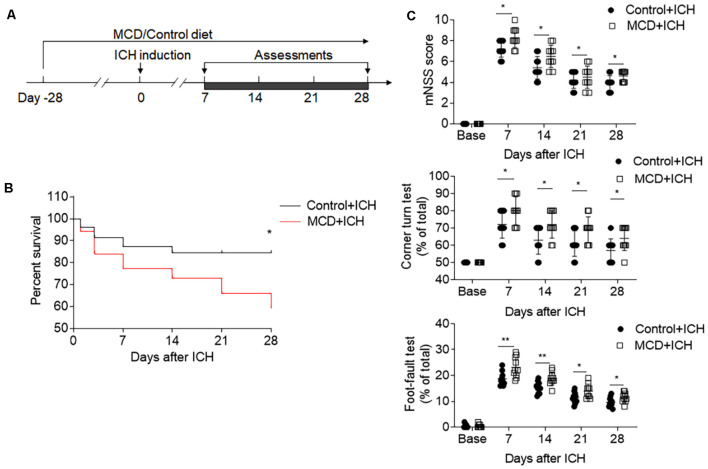
NAFLD led to the worse neurological outcome and increased mortality after ICH. ICH was induced in mice by injection of collagenase. A battery of neurological tests was conducted to assess the motor, sensory, and balance functions in two groups of mice at days 7, 14, 21, and 28 after ICH. **(A)** Flow chart illustrates the individual diet administration and experimental design. **(B)** The long-term survival of ICH mice with control or MCD diet after ICH. **(C)** NAFLD mice had more serious neurological function loss evaluated by mNSS test, corner turning test, and foot-fault test at days 7, 14, 21, and 28 after ICH. *n* = 10 mice per group. Data are presented as mean ± SD. *P < 0.05, **P < 0.01.

We also evaluated the effects of NAFLD on the outcome of ICH at later time points. The results of mNSS, corner test, and foot fault test show that the detrimental effects of NAFLD on ICH persisted until at least 28 days after ICH induced by collagenase injection (Figures 2A,C). Furthermore, mice fed with NAFLD also have decreased survival index (Figure 2B). These results suggest that NAFLD could impair the outcome after ICH.

### NAFLD Augments Brain Inflammation Characterized by Increased Myeloid Cell Infiltration and MMP9 Production in the Brain After ICH

**Figure 3:**
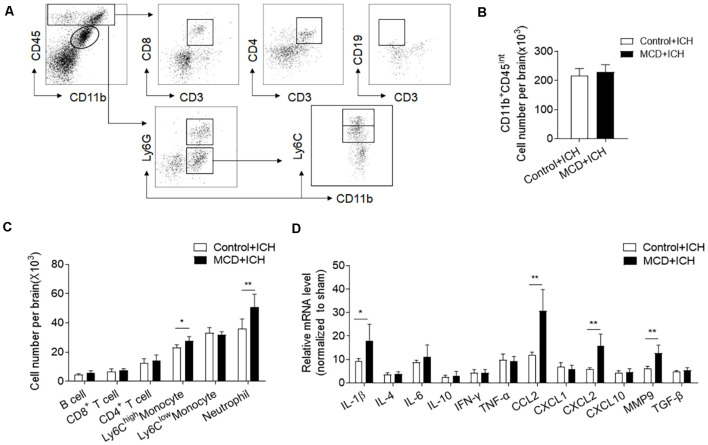
NAFLD augments leukocyte infiltration and local inflammation in the brain after ICH. On day 3 after ICH induction by collagenase injection in NAFLD mice or control mice, the counts of immune cell subsets in the brain were measured by FACS on day 3 after ICH. **(A)** The gating strategy for microglia (CD11b^+^CD45^int^), CD8^+^ T cells (CD45^high^CD3^+^CD8^+^), CD4^+^ T cells (CD45^high^CD3^+^CD4^+^), B cells (CD45^high^CD3^−^CD19^+^), Neutrophils (CD45^high^CD11b^+^Ly6G^+^), proinflammatory monocytes (CD45^high^CD11b^+^ Ly6G^−^ Ly6C^high^) cells. **(B,C)** Bar graph illustrates the counts of indicated cell subsets in brains from ICH mice. *n* = 6 per group. **(D)** The bar graph shows the expression of cytokines and chemokines in the ipsilateral hemispheres including IL-1β, IL-4, IL-6, IL-10, IFN-γ, TNF-α, CCL2, CXCL1, CXCL2, CXCL10, MMP9, and TGF-β, *n* = 6 mice per group. Data are presented as mean ± SD. *P < 0.05, **P < 0.01.

Inflammation is a key factor causing secondary brain injury after ICH (Wang, 2010). We thus determined whether NAFLD has an impact on brain inflammation after ICH. Using flow cytometry, we examined the counts of infiltrating leukocytes as well as brain resident microglia at days 3 after ICH. The results showed that the counts of brain-infiltrating neutrophils (CD45^high^CD11b^+^Ly6G^+^) and monocytes (CD45^high^CD11b^+^Ly6G^−^Ly6C^high^) were significantly increased in NAFLD mice compared to control mice at day 3 after ICH, but microglia and lymphocytes including T cells and B cells were not significantly different between these two groups (Figures 3A–C). Besides, RT-PCR data show that NAFLD also increased the mRNA levels of IL-1β, CCL2, CXCL2, and MMP9 in the ipsilateral hemisphere after ICH, most of these genes are related to myeloid cell infiltration and function (Figure 3D).

**Figure 4:**
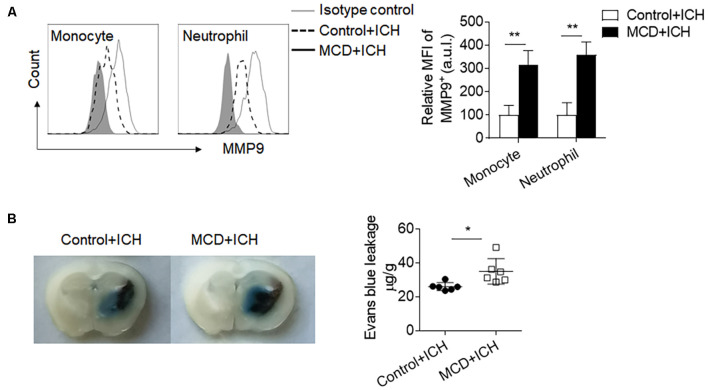
NAFLD augmented MMP9 production and blood-brain barrier (BBB) disruption after ICH. ICH was induced by collagenase injection in NAFLD mice and control mice. MMP9 expressed by myeloid cells in mice brains was assessed at day 3 after ICH induction. **(A)** The mean fluorescence intensity of MMP9 in neutrophils and monocytes was quantified by flow cytometry. *n* = 6 per group. **(B)** Histological images show that NAFLD increased Evans blue dye leakage at day 3 after ICH. *n* = 6 mice per group. Data are presented as mean ± SD. *P < 0.05, **P < 0.01.

Myeloid cells are the primary leukocyte subset to infiltrate the ICH brain and boost local inflammation by the production of pro-inflammatory factors. Among these factors, MMP9 is a pivotal contributor to BBB disruption. We found that the MFI (Mean Fluorescence Intensity) values of MMP9 in the brain-infiltrating neutrophils and monocytes were significantly increased in ICH mice receiving the MCD diet (Figure 4A). Besides, the extravasation of Evans blue dye was also aggravated in ICH mice receiving the MCD diet (Figure 4B). Together, these results indicate that NAFLD facilitates brain infiltration of myeloid cells and local inflammation, which may contribute to exacerbated ICH injury.

### NAFLD Mobilizes Neutrophils and Monocytes in the Periphery Before ICH

**Figure 5:**
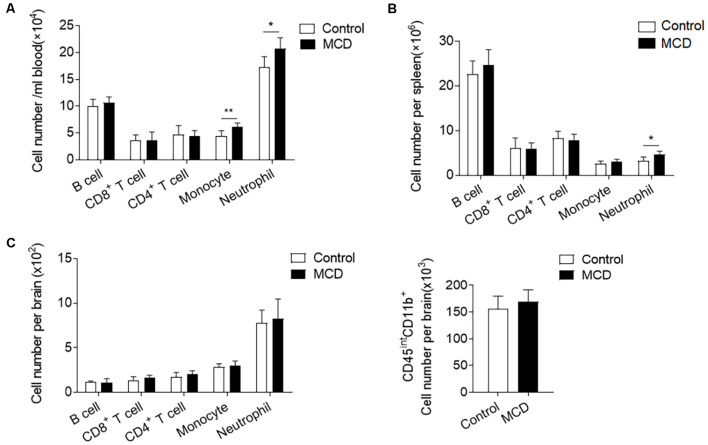
NAFLD enhanced the mobilization of myeloid cells in the periphery. Immune cells were isolated from circulating blood, spleen, and brain tissues of NAFLD mice or control mice. **(A,B)** Summarized results of the counts of neutrophil (CD11b^+^Ly6G^+^), Ly6C^high^ monocytes (CD11b^+^Ly6G^−^Ly6C^high^), CD4^+^ T cells (CD3^+^CD4^+^), CD8^+^ T cells (CD3^+^CD8^+^) and B cells (CD3^−^CD19^+^) in blood **(A)** and spleen **(B)** of NAFLD mice and control mice. *n* = 6 per group. **(C)** Summarized results of the counts of neutrophil (CD45^high^CD11b^+^Ly6G^+^), pro-inflammatory monocytes (CD45^high^CD11b^+^Ly6G^−^Ly6C^high^), CD4^+^ T cells (CD45^high^CD3^+^CD4^+^), CD8^+^ T cells (CD45^high^CD3^+^CD8^+^), B cells (CD45^high^CD3^−^CD19^+^) and microglia (CD45^int^CD11b^+^) in brain of NAFLD mice and control mice. *n* = 6 per group. Data are presented as mean ± SD. *P < 0.05, **P < 0.01.

To investigate the influence of NAFLD on immune responses, we compared the counts of immune cell subsets in spleens, circulating blood, and brains of NAFLD mice and control mice before ICH. Flow cytometric analysis revealed that the number of neutrophils (CD11b^+^Ly6G^−^Ly6C^high^) was significantly increased in the circulating blood and spleen from the mice with the MCD diet as compared to the control diet (Figures 5A,B). Pro-inflammatory monocytes (CD11b^+^Ly6C^high^) were also obviously increased in the blood (Figure 5A). In contrast, other immune cells including the counts of CD4^+^ T cells (CD3^+^CD4^+^), CD8^+^ T cells (CD3^+^CD8^+^), and B cells (CD3^−^CD19^+^) were not significantly altered in the spleen or circulating blood (Figures 5A,B). It is noteworthy that NAFLD did not have an impact on leukocyte infiltration or the number of microglia in the brain before ICH (Figure 5C). Our results indicate that NAFLD can mobilize neutrophils and monocytes in the periphery before ICH, which may enhance the mobilization of these cells into the brain after ICH.

### Depletion of Myeloid Cells Alleviated NAFLD-Induced Exacerbation of Hemorrhagic Brain Injury

**Figure 6:**
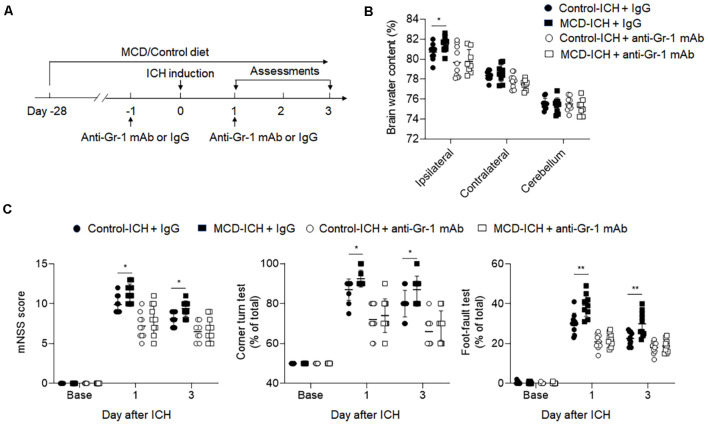
The detrimental effect of NAFLD was abolished in ICH mice depleted of myeloid cells. **(A)** Flow chart illustrates drug administration and experimental design. NAFLD or control mice were given anti-Gr-1 mAb or IgG 1 day before and 1 day after ICH. Before ICH and at days1 and 3 after ICH, neurological deficits and brain water content were evaluated. **(B)** Results of the brain water content of mice receiving indicated treatments at day 3 after ICH. *n* = 10 per group. **(C)** Statistical data of neurological deficits assessment including mNSS test, corner-turning test, and foot-fault test score in indicated groups of mice. *n* = 10 per group. Data are presented as mean ± SD. *P < 0.05, **P < 0.01.

Based on our previous data, we focused on studying the role of myeloid cells in the detrimental effects of NAFLD on ICH. We applied an anti-Gr-1 mAb (RB6-8C5) to deplete Gr-1^+^ myeloid cells, including neutrophils and monocytes that are known participants in brain inflammation and BBB disruption (Hammond et al., 2014). Drug administration and experimental design were shown in Figure 6A. The efficacy of Gr-1^+^ depletion was about 90% (Supplementary Figure S2). We found the aggravation of hemorrhagic severity by NAFLD was much alleviated in the condition of Gr-1^+^ cell depletion, displaying decreased brain water content and neurological deficit scores (Figures 6B,C). These results demonstrated that neutrophils and monocytes are involved in the detrimental effects of NAFLD on hemorrhagic brain injury.

## Discussion

This study provides the first evidence that NAFLD exacerbates hemorrhagic brain injury in two mouse models of ICH. As documented here, NAFLD not only aggravates neurological deficits and brain edema in the acute stage of ICH but also leads to persistent neurological dysfunction and reduced survival index. NAFLD augments leukocyte infiltration and the production of proinflammatory factors in the ICH brain. Importantly, we found that NAFLD mobilizes peripheral neutrophils and monocytes before ICH induction, suggesting that NAFLD induces mobilization of the peripheral immune system. Also, results from antibody-mediated depletion of neutrophils and monocytes revealed the contribution of these cells to the detrimental effects of NAFLD on ICH.

Emerging evidence has demonstrated that brain inflammation exacerbates secondary brain injury and brain edema development after ICH (Wang, 2010). Neutrophils and monocytes are among early responders to ICH and migrate to the injury site at a very early stage of ICH, leading to augmented local inflammation by producing a burst of pro-inflammatory factors. As previously documented, neutrophils and monocytes were observed in and around the hematoma as early as 4–5 h and peaked at 3 days after ICH onset (Wang and Tsirka, 2005; Wang and Dore, 2007). Reportedly, depletion of neutrophils in ICH animals reduced blood-brain barrier (BBB) breakdown, inflammation, and MMP-9 expression (Moxon-Emre and Schlichter, 2011). Consistent with these previous findings, we found that NAFLD increased MMP-9 production by brain-infiltrating neutrophils and monocytes that may contribute to the BBB disruption and perihematomal edema. These results demonstrate that NAFLD augments brain inflammation after ICH, and highlight the involvement of neutrophils and monocytes in such processes.

NAFLD patients were found to have a persistent increase of circulating neutrophils and monocytes (Zhang et al., 2018; Khoury et al., 2019). As an extension of these previous findings, we found that mobilization of neutrophils and monocytes in the periphery contributes to the aggravation of hemorrhagic injury in NAFLD mice. These results support the notion that neutrophils and monocytes are involved in the detrimental effects of NAFLD in ICH injury. It’s also noteworthy that the number and activity of microglia were not affected by NAFLD, suggesting that NAFLD-induced mobilization of immune responses likely only occurs in the peripheral compartment rather than the brain, at least in our experimental setting. Together, our findings provide novel evidence regarding NAFLD-induced mobilization of neutrophils and monocytes, although the exact operating mechanisms through which NAFLD mobilizes neutrophils and monocytes and the subsets of these myeloid cells (i.e., neutrophils or monocytes) that is particularly involved in the pathological mechanisms of NAFLD to exacerbate ICH injury requires future investigation.

Changes in lifestyle and resultant over-nutrition are common causes of NAFLD (Farrell et al., 2013). More than 25% of NAFLD patients develop potential nonalcoholic steatohepatitis (NASH) characterized by lipid accumulation, liver cell injury, and inflammation (Singh et al., 2015). In our study, we fed mice with an MCD diet for 4 weeks to induce NAFLD and hyperlipidemia. This is a well-established model to mimic human NAFLD features including hyperlipidemia, steatosis, inflammation, and fibrosis (Yu et al., 2010). Notably, we found unaltered coagulation time in NAFLD mice receiving MCD diet, suggesting that MCD diet-induced liver injury may not influence the coagulation or clotting processes, at least in the current experimental setting. Also, we are aware that the exacerbation of ICH injury in NAFLD animals cannot be entirely attributed to NAFLD-induced mobilization of peripheral neutrophils and monocytes. This is because NAFLD can also elicit other pathological events such as cerebrovascular alterations that may contribute to the predisposition of ICH injury. In this regard, these possibilities warrant future studies. Also, only male mice were used in this study. Future studies should also include female mice to clarify if there are any effects of sex differences in NAFLD-induced aggravation of ICH injury.

In summary, our findings suggest that the mobilization of neutrophils and monocytes in NAFLD mice contributes to aggravated hemorrhagic brain injury. We, therefore, infer that the prevention and control of NAFLD may reduce the detrimental consequences of ICH.

## Conclusion

We demonstrate that NAFLD worsens neurological deficits and brain injury after ICH, accompanied by augmented brain infiltration of neutrophils and monocytes and the production of pro-inflammatory factors. Our data suggest that the prevention and control of NAFLD would reduce the detrimental consequences of ICH.

## Data Availability Statement

All datasets generated for this study are included in the article/Supplementary Material.

## Ethics Statement

All animal experiments were reviewed and approved by the Institutional Animal Care and Use Committees of Tianjin Medical University General Hospital (Tianjin, China). This study was conducted following the National Institutes of Health Guide for the Care and Use of Laboratory Animals in China.

## Author Contributions

ML, QL, and HM formulated the concept and designed the study. XL and XC performed the studies. XL and XW analyzed the data and interpreted the results. ML, QL, and XL wrote the article.

## Conflict of Interest

The authors declare that the research was conducted in the absence of any commercial or financial relationships that could be construed as a potential conflict of interest.

## Abbreviations

NAFLD: Non-alcoholic fatty liver disease
ICH: Intracerebral hemorrhage
MCD: Methionine choline-deficient
mNSS: Modified neurological severity score
MMP-9: Matrix metalloproteinase-9
PCR: Polymerase chain reaction
MFI: Mean fluorescence intensity
BBB: Brain blood barrier
NASH: Nonalcoholic steatohepatitis.
