# Supplementary material for: Dyslipidemic Diet Induces Mobilization of Peripheral Neutrophils and Monocytes That Exacerbate Hemorrhagic Brain Injury and Neuroinflammation
Source: Front Cell Neurosci. 2020 Jun 8;14:154. doi: 10.3389/fncel.2020.00154 (PMC7325918; doi:10.3389/fncel.2020.00154)
Supplement: Supplementary file 1 [file Data_Sheet_1.docx]

**Supplementary Figure Legends**


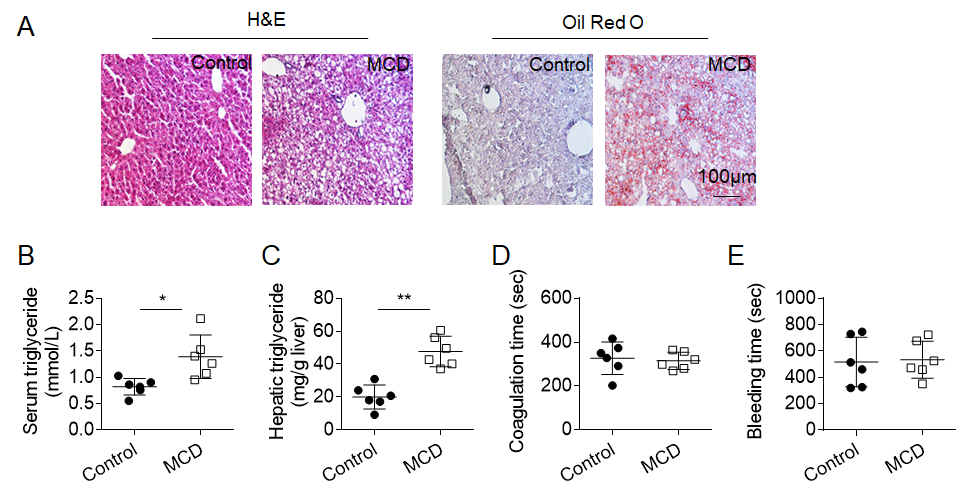


**Supplemental Figure 1.** The NAFLD mice exhibited fat accumulation and increased triglycerides (TG) in liver, while not affecting coagulation and bleeding time. C57BL/6 mice were fed a methionine-choline deficient diet (MCD) for 4 weeks to induce NAFLD model. **A.** Images of H＆E and Oil Red O staining in liver from mice receiving MCD diet or control diet. The scale bar represents 100 μm. **B-C.** Quantification of triglycerides (TG) from serum and liver samples from NAFLD mice and control mice. n=6 per group. **D-E.** The results of coagulation time and bleeding time between the indicated groups revealed no obvious change. n=6 per group. Data are presented as mean ± SD. **P*<0.05, ***P*<0.01.


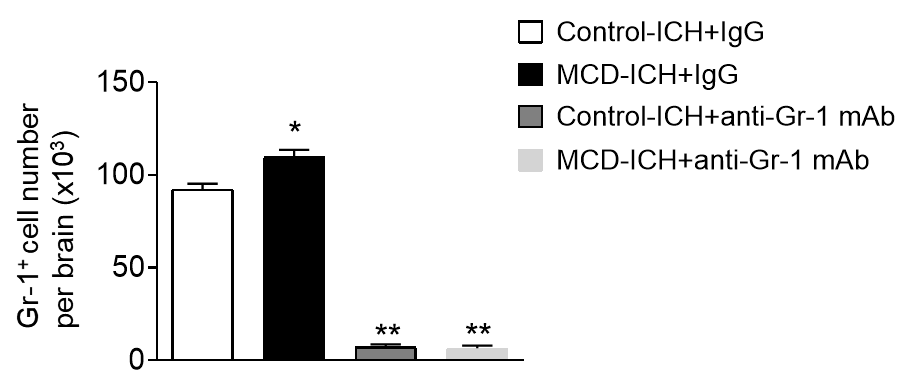


**Supplemental Figure 2.** **Counts of Gr-1^+^ cells in the ICH brain of mice with the indicated treatment at day 3 after ICH.** C57BL/6 mice was fed with MCD diet or control diet for 4 weeks, then receiving anti-Gr-1 antibody or IgG injection. At day 3 after ICH, brain tissue were harvested for flow cytometry analysis. n = 6 mice per group. Data are presented as mean ± SD. **P*<0.05, **P*<0.01 vs. the group of Control-ICH+IgG.

**Supplemental Table 1. List of** **primer sequences used for RT-PCR analysis in this study.**

| Genes | Primers (Forward, 5’-3’) | Primers (Reverse, 5’-3’) |
| --- | --- | --- |
| GAPDH | GCCAAGGCTGTGGGCAAGGT | TCTCCAGGCGGCACGCAGA |
| IL-1β | TGCCACCTTTTGACAGTGATG | TGATGTGCTGCTGCGAGATT |
| IL-4 | TTGTCATCCTGCTCTTCTTTCT | CTGTGGTGTTCTTCGTTGCT |
| IL-6 | GCTGGTGACAACCACGGCCT | AGCCTCCGACTTGTGAAGTGGT |
| IL-10 | AAATAAGAGCAAGGCAGTGG | GTCCAGCAGACTAAATACACAC |
| TNF-α | TATG GCTCAGGGTCCAACTC | GGAAAGCCCATTTGAGTCCT |
| CCL2 | CTGCTGTTCACAGTTGCCG | GCACAGACCTCTCTCTTGAGC |
| CXCL1 | CTTGCCTTGACCCTGAAGTCT | AGCAGTCTGTCTTCTTTCTCCGT |
| CXCL2 | AGGGCGGTCAAAAAGTTTGC | CGAGTACGATCCAGGCTTGC |
| CXCL10 | AAGCTATGTGGAGGTGCGAC | AACCCCTTGGGAAGATGGTG |
| MMP9 | CATTCGCGTGGATAAGGAGT | ACCTGGTTCACCTCATGGTC |
| IFN-γ | CTGCTGATGGGAGGAGATGT | TTTGTCATTCGGGTGTAGTCA |
| TGF-β | TGCGCTTGCAGAGATTAAAA | CGTCAAAAGACAGCCACTCA |
